# Management of postoperative atrial fibrillation

**DOI:** 10.1007/s00540-012-1330-9

**Published:** 2012-01-25

**Authors:** Takeshi Omae, Yuichi Kanmura

**Affiliations:** 1 Department of Anesthesiology, Fujimoto Hayasuzu Hospital, 17-1 Hayasuzu, Miyakonojo, Miyazaki 885-0055 Japan; 2 Departments of Anesthesiology and Critical Care Medicine, Kagoshima University, Graduate School of Medical and Dental Sciences, Kagoshima, Kagoshima Japan

**Keywords:** Atrial fibrillation, Cardiovascular surgery, Statins, Rate control, Anticoagulant therapy

## Abstract

The impact of postoperative atrial fibrillation (PAF) on patient outcomes has prompted intense investigation into the optimal methods for prevention and treatment of this complication. In the prevention of PAF, β-blockers and amiodarone are particularly effective and are recommended by guidelines. However, their use requires caution due to the possibility of drug-related adverse effects. Aside from these risks, perioperative prophylactic treatment with statins seems to be effective for preventing PAF and is associated with a low incidence of adverse effects. PAF can be treated by rhythm control, heart-rate control, and antithrombotic therapy. For the purpose of heart rate control, β-blockers, calcium-channel antagonists, and amiodarone are used. In patients with unstable hemodynamics, cardioversion may be performed for rhythm control. Antithrombotic therapy is used in addition to heart-rate maintenance therapy in cases of PAF >48-h duration or in cases with a history of cerebrovascular thromboembolism. Anticoagulation is the first choice for antithrombotic therapy, and anticoagulation management should focus on maintaining international normalized ratio (INRs) in the 2.0–3.0 range in patients <75 years of age, whereas prothrombin-time INR should be controlled to the 1.6–2.6 range in patients ≥75 years of age. In the future, dabigatran could be used for perioperative management of PAF, because it does not require regular monitoring and has a quick onset of action with short serum half-life. Preventing PAF is an important goal and requires specific perioperative management as well as other approaches. PAF is also associated with lifestyle-related diseases, which emphasizes the ongoing need for appropriate lifestyle management in individual patients.

## Introduction

Postoperative atrial fibrillation (PAF) is the most frequent complication that occurs after cardiovascular surgery (Table 1) [1]. The incidence of cardiovascular events, such as cerebral infarction and heart failure, increases by twofold in the presence of chronic atrial fibrillation (AF) [2]. Conventional viewpoints suggest that PAF is less likely to affect the survival of patients when compared with chronic AF, although it does slightly prolong the duration of hospital stay. However, reports suggest that PAF is associated with a significant incidence of various complications, including cardiovascular events, renal failure, infection, and cerebral infarction [3]. PAF is affected by diverse factors, and a wide variety of strategies has been shown to be useful in preventing or treating PAF. This article outlines the etiology, characteristics, and PAF prevention and treatment, citing the latest findings from published reports.

**Table 1:** Postoperative complications after coronary artery bypass grafting

Complication	Percent of patients
Rethoracotomy	2
Renal failure	5
Cerebral infarction	2.5
Respiratory failure	6
Gastrointestinal failure	2
Atrial fibrillation	30

## PAF characteristics

AF is the most frequent complication arising after coronary artery bypass (CAB) surgery, occurring in 30% of cases. The incidence of this complication is even higher after valve-replacement surgery (30–40%) and after compound operative procedures (40–60%) [4]. The onset of AF has also been reported after nonheart surgery, such as pulmonary lobectomy (10–20%) and total pneumonectomy (40%) [5]. PAF most frequently develops on the second postoperative day. Although often transient, it recurs in 40% of cases [3]. Historically, in contrast to chronic AF, PAF was thought to be relatively unlikely to affect patient survival. However, more recent studies suggest that PAF increases the duration of intensive care unit (ICU) stay (2.0 days in the no-PAF group vs. 3.6 days in the PAF group, *p* < 0.001), duration of hospital stay (7 days in the no-PAF group vs. 10 days in the PAF group, *p* < 0.001), and is associated with a twofold elevation in the incidence of cerebral infarction (2.4% in the no-PAF group vs. 5.3% in the PAF group, *p* < 0.001) and a increased risk of 30-day mortality (3.0% in the no-PAF group vs. 6.0% in the PAF group, *p* < 0.001). Furthermore, PAF was an independent predictor of long-term mortality [adjusted odds ratio (OR) 1.5, *p* < 0.001] in a retrospective cohort; OR 3.4, *p* < 0.0018 in a case-matched population. [4]. Thus, PAF appears to have a significant effect on the acute postoperative condition of patients as well as on their long-term prognosis.

Preoperative factors associated with an increased risk of developing PAF are summarized in Table 2. The incidence of PAF is particularly high among elderly patients [4, 6, 7], especially in those >70 years of age [8]. Conventional risk factors for PAF include left atrial dilatation and left ventricular hypertrophy [7, 8]; other risk factors include diabetes mellitus [7, 8], obesity, and metabolic syndrome [9]. A recent study reported that obesity is an independent risk factor for PAF in those undergoing cardiovascular surgery, especially in patients >50 years of age [10, 11]. Intraoperative risk factors for PAF are summarized in Table 3 and include atrial injury, atrial ischemia, insertion of a vascular catheter, and sudden changes in circulating blood volume [7–9]. Furthermore, cardiovascular surgery using cardiopulmonary bypass is likely to induce an inflammatory reaction, triggering the onset of PAF [12]. Postoperative risk factors for PAF are summarized in Table 4 and include volume overload, electrolyte abnormalities, atrial extrasystole, and sympathetic hyperactivity [10]. In addition, studies suggest that inflammatory reactions are a risk factor for PAF [13, 14]. Delineation of these novel risk factors for PAF has led to the development of new strategies for treating and preventing PAF.

**Table 2:** Preoperative risk factors for postoperative atrial fibrillation

Risk factors	
Old age	Diabetes
Enlargement of the left atrium	Obesity
Left ventricular hypertrophy	Metabolic syndrome
Hypertension

**Table 3:** Intraoperative risk factors for postoperative atrial fibrillation

Risk factors	
Damage to the atrium	Insertion of a ventilator tube
Myocardial ischemia	Venous cannulation
Acute volume change

**Table 4:** Postoperative risk factors for postoperative atrial fibrillation

Risk factors	
Volume overload	Atrial extrasystole
Increased afterload	Imbalance of the autonomic nervous system
Hypotension	Electrolyte imbalance
Inflammation	

## Preventing PAF

Conventionally, nondihydropyridine calcium-channel antagonists and digitalis have been used for treating PAF [15, 16]. Although nondihydropyridine calcium-channel antagonists are effective against supraventricular tachycardia [17], they are also frequently associated with adverse reactions (e.g., atrioventricular block, heart failure, etc.) [18] and are therefore not the ideal agents to use for prophylactic purposes. Further, one meta-analysis reported that digitalis was ineffective when used for PAF prevention. This is probably because digitalis acts on vagal tone and therefore reduces the ventricular rate during atrial arrhythmias. Therefore, this benefit of digitalis may be attenuated during the perioperative period, in which there is increased sympathetic tone [19]. Methods reported to be useful in PAF prevention are summarized in Table 5.

**Table 5:** Prevention of postoperative atrial fibrillation

Preventive measures	
β-blockers	Steroids
Amiodarone	Statins
OPCAB	Pacing

*OPCAB* off-pump coronary artery bypass grafting

### β-blockers

β-blockers have direct antiarrhythmic activity on stimulus conduction cells and myocardial cells and are classified as class II antiarrhythmic agents according to the Vaughan–Williams classification. β-blockers are effective against tachycardic arrhythmia [20], supraventricular arrhythmia [21], and lidocaine-resistant ventricular fibrillation [22]. Perioperative prophylactic use of β-blockers could decrease cardiovascular events [23–31]. Coleman et al. [32] reported that postoperative β-blockers reduced hospital length of stay [mean ± standard deviation (SD) 10.22 ± 11.38 in the β-blocker group vs. 12.40 ± 15.67 in the placebo group; *p* = 0.001] and PAF (23.5% in the β-blocker group vs. 28.4% in the placebo group; *p* = 0.02). Crystal et al. [33] reported that β-blockers had the greatest magnitude of effect for PAF prevention in a meta-analysis [OR 0.35; 95% confidence interval (CI) 0.26–0.49]. Lindenauer et al. [34] reported that prophylactic β-blockers were associated with a reduced risk of in-hospital death in patients with a Revised Cardiac Risk Index (RCRI, which is based on the presence of history of ischemic heart disease, cerebrovascular disease, renal insufficiency, diabetes mellitus, or a patient undergoing high-risk surgery) of score ≥3 but not in patients with a RCRI score of 2, l, or 0. Thus, that study demonstrated an association between improved outcomes and the use of β-blockers in clinically high-risk patients, whereas lower-risk patients had worse outcomes. The PeriOperative Ischemia Study Evaluation (POISE) trial [35], which was a large, randomized, controlled trial of fixed, high-dose, extended-release metoprolol starting on the day of surgery in more than 8,000 patients undergoing noncardiac surgery, demonstrated that perioperative β-blocker treatment reduced the incidence of cardiovascular events [5.8% patients in the metoprolol group vs. 6.9% patients in the placebo group; hazard ratio (HR) 0.84; 95% CI 0.70–0.99; *p* = 0.04] but increased the total death rate (3.1 vs. 2.3%; HR 1.33, 95% CI 1.03–1.74; *p* = 0.03) or the incidence of stroke (1.0 vs. 0.5%; HR 2.17; 95% CI 1.26–3.74; *p* = 0.005), possibly due to β-blocker-induced hypotension (15 vs. 9.7%; HR 1.55; 95% CI 1.38–1.74) and bradycardia (6.6 vs. 2.4%; HR 2.74; 95% CI 2.19–3.43). Further, a meta-analysis of data from 33 randomized controlled trials (RCTs) reported that β-blocker treatment reduced nonfatal myocardial infarction (MI) (OR 0.65; 95% CI 0.54–0.79) and myocardial ischemia (OR 0.36; 95% CI 0.26–0.50) but increased nonfatal strokes (OR 2.01; 95% CI 1.27–3.68) and the incidence of bradycardia (OR 3.13; 95% CI 2.51–3.92) and hypotension (OR 1.69; 95% CI 1.39–2.65) that required treatment [36]. These data suggest that prophylactic β-blockers should be used with caution. Guidelines for using β-blockers were updated after results from the POISE trial became available. At present, unless contraindicated, treatment with β-blockers is recommended to prevent PAF [Class I, level of evidence (LOE) A; Table 6] in the American College of Cardiology (ACC)/American Heart Association (AHA)/European Society of Cardiology (ESC) Guidelines for the Management of Patients with Atrial Fibrillation [37]. Further, preoperative or early postoperative administration of β-blockers in patients without contraindications should be used as standard therapy to reduce the incidence and/or clinical sequelae of AF and is recommended in the ACC/AHA Guideline Update for Coronary Artery Bypass Graft Surgery (Class I, LOE B; Table 6) [38]. The POISE trial did not study continuation of β-blockers in patients undergoing surgery who are receiving β-blockers for the ACC/AHA Class I guideline indications. The guidelines on perioperative cardiovascular evaluation and care for noncardiac surgery also state that β-blockers should be continued in patients undergoing surgery who are receiving β-blockers for treatment of conditions with ACC/AHA Class I guideline indications for the drugs (Class I, LOE C; Table 6) [39].

**Table 6:** Classification of recommendations

ACC/AHA/ESC guideline recommendations	
Class	
Class I	Conditions for which there is evidence and/or general agreement that a given procedure/therapy is beneficial, useful, and effective
Class II	Conditions for which there is conflicting evidence and/or a divergence of opinion about the usefulness/efficacy of performing the procedure/therapy
Class IIa	Weight of evidence/opinion is in favor of usefulness/efficacy
Class IIb	Usefulness/efficacy is less well established by evidence/opinion
Class III	Conditions for which there is evidence and/or general agreement that a procedure/therapy is not useful or effective and in some cases may be harmful
Level of evidence	
Level of evidence A	Data derived from multiple randomized clinical trials or meta-analyses
Level of evidence B	Data derived from a single randomized trial, or nonrandomized studies
Level of evidence C	Only consensus opinion of experts, case studies, or standard of care

Classification of recommendations and level of evidence are expressed in the ACC/AHA/ESC format. Recommendations are evidence based and derived primarily from published data

Weight of evidence is ranked from highest (A) to lowest (C)

*ACC* American College of Cardiology, *AHA* American Heart Association, *ESC* European Society of Cardiology

### Amiodarone

Amiodarone is a multichannel blocker possessing α, β, potassium (K^+^) channel, sodium (Na^+^) and calcium (Ca^2+^)-blocking actions. It is a Class III antiarrhythmic agent according to the Vaughan–Williams classification. Amiodarone regimens are used to prevent or treat PAF. In one report, a 1-week preoperative oral regimen decreased the incidence of PAF (25% patients in the amiodarone group vs. 53% patients in the placebo group, *p* = 0.03) [40], postoperative intravenous treatment decreased the incidence of PAF when compared with placebo (35% patients in the amiodarone group vs. 47% patients in the placebo group, *p* = 0.01) [41], and treatment throughout the perioperative period decreased the incidence of PAF when compared with placebo (16% patients in the amiodarone group vs. 25% patients in the placebo group, *p* = 0.001) [42]. Amiodarone is also recommended as an appropriate means of preventing AF in high-risk patients (LOE A, Class IIa; Table 6) [37]. However, a meta-analysis of randomized placebo-controlled trials showed that perioperative amiodarone treatment was associated with an increase in the incidence of adverse reactions (1.7-fold in bradycardia and 1.6-fold in hypotension) [43]. Risk factors for amiodarone-related adverse reaction included intravenous administration, and administration of >1 g per day [43]. In view of these findings, it is advisable to avoid prophylactic amiodarone treatment in patients at low risk for developing PAF. In those who do require prophylactic treatment, clinicians should be aware of the potential for adverse reactions (bradycardia, hypotension, etc.) during the use of this drug. β-blockers and amiodarone are particularly effective for perioperative prophylactic treatment and are recommended for this purpose in the Medical Society guidelines [37–39]. Otherwise, clinicians should be cognizant that there is a risk of drug-related adverse reactions, particularly bradycardia and hypotension, when using these drugs.

### Statins

Autopsy studies have demonstrated the role of plaque rupture and erosion in the pathophysiology of acute coronary syndrome. Statins have pleiotropic action, including the modification of atherosclerotic plaques and the improvement of endothelial function. Inflammation plays a role in the pathogenesis of atherosclerosis, and the anti-inflammatory properties of stains likely account for their ability to reduce the incidence of PAF and perioperative cardiovascular events [44, 45]. Mariscalco et al. reported that PAF occurred in 29.5% of patients receiving preoperative statin therapy when compared with 40.9% of patients who were not receiving such therapy (*p* = 0.021). Preoperative statins were associated with a 42% reduction in the risk of AF development after CAB surgery (OR 0.58; 95% CI 0.37–0.91, *p* = 0.017, stratified by propensity score) [46]. A meta-analysis of randomized trials of the use of preoperative statins reported that statins significantly reduced postoperative MI [risk ratio (RR) 0.57; CI 0.46–0.70; *p* < 0.0001], and PAF (RR 0.54; 95% CI 0.43–0.68; *p* < 0.0001) [47]. Guidelines do not specifically recommend the use of statins before cardiovascular surgery for the express purpose of preventing periprocedural complications, such as PAF. However, at present, perioperative prophylactic treatment with statins that have few side effects seems to be appropriate for PAF prevention of PAF.

### Anti-inflammatory drugs

Cardiovascular surgery with cardiopulmonary bypass is associated with a systemic inflammatory response, which may be in part responsible for PAF. Patients with PAF tend to have significantly higher C-reactive protein (CRP), higher white blood cell (WBC) counts, and higher levels of inflammatory cytokines when compared with patients who do not develop PAF [48, 49]. This finding suggests that inflammatory reactions may be important in the pathogenesis of PAF [48, 49]. In nonsurgical patients, corticosteroid treatment may reduce the incidence of recurrent AF. Halonen et al. [50] reported that the administration of 100 mg of hydrocortisone reduced PAF when compared with placebo in a randomized control trial (30% in the 100-mg hydrocortisone group vs. 48% in the placebo group; adjusted HR 0.54; 95% CI 0.35–0.83, *p* = 0.004). Corticosteroids have anti-inflammatory properties. Several studies have indicated that the postoperative concentration of CRP was significantly lower in patients who received hydrocortisone than in patients who did not. Corticosteroids also reduce postoperative nausea, vomiting, and anorexia. Thus, corticosteroid therapy may improve absorption of orally administered medications, such as β-blockers, and thereby reduce the incidence of PAF. Cheruku et al. administrated 30 mg ketorolac intravenously every 6 h until patients were able to take medications orally; then, patients were switched to ibuprofen 600 mg orally three times a day for a total of 7 days or until discharge, whichever was longer [nonsteroidal anti-inflammatory drug (NSAID) group]. The authors reported that NSAIDs reduced the incidence of PAF (9.8% vs. 28.6% in the placebo group) [51]. However, NSAIDs are associated with nephrotoxicity, particularly when used in the postoperative period, and in elderly patients.

### Magnesium

Some studies have reported that serum magnesium levels are low in patients who develop PAF [52] and that tachycardia arrhythmia is closely related to the magnesium level [53]. A meta-analysis demonstrated that magnesium administration decreased the proportion of patients developing postoperative AF from 28% in the control group to 18% in the treatment group (OR 0.54; 95% CI 0.38–0.75) [54]. In another meta-analysis, magnesium prevented PAF (OR 0.57; 95% CI 0.42–0.77), but there was significant heterogeneity (*p* < 0.001) [29]. These meta-analyses included a small number of patients, and the design varied among the different studies, which limits the interpretation of the results. Although there are some data to suggest that perioperative administration of magnesium can prevent PAF in a manner similar to perioperative treatment with antiarrhythmic drugs, this notion remains controversial.

### Off-pump coronary artery bypass grafting (OPCAB)

CAB surgery can be divided into procedures that use extracorporeal circulation and those that do not (OPCAB). OPCAB requires less manipulation of the aorta and avoids the use of cardiopulmonary bypass and may therefore cause less postoperative complications [55]. The incidence of PAF is lower following OPCAB when compared with conventional CAB surgery using cardiopulmonary bypass (OR 0.78; 95% CI 0.74–0.82; *p* < 0.0001 in an observational study; OR 0.59; 95% CI 0.46–0.77; *p* < 0.0001 in a randomized controlled study) [55]. However, revascularization procedures did not affect postoperative mortality or stroke rates in a recent cohort study [56] and did not affect the incidence of PAF in a recent randomized trial [57]. In high-risk patients, Moller et al. [58] reported that the incidence of cardiovascular events, including PAF, was similar when comparing on-pump and off-pump revascularization. Thus, it remains controversial whether or not OPCAB reduces the incidence of PAF.

### Atrial pacing

Prophylactic atrial pacing to prevent PAF after cardiovascular surgery is based on the fact that pacing is thought to favorably influence intra-atrial conduction and atrial refractoriness. There are several mechanisms by which atrial pacing might prevent AF: reduction of the bradycardia-induced dispersion of atrial repolarization, avoidance of the trigger for AF by overdrive suppression, and changing atrial activation pattern by dual-site atrial pacing [10]. Meta-analyses have demonstrated that single-site atrial pacing and biatrial pacing can reduce the incidence of PAF [29, 33, 59]. Fan et al. [60] compared these two methods of atrial pacing and reported that biatrial pacing was superior in terms of maintaining interatrial conduction and is thus more useful in terms of preventing PAF than single-site atrial pacing. However, the number of enrolled patients was small, and the protocols varied widely among studies. Indeed, a major adverse effect of prophylactic atrial pacing is its potential proarrhythmic effect, which might be precipitated by inappropriate sensing or loss of pacing through temporary wires. Further, biatrial pacing requires complex manipulations, which has limited its use as a means of preventing AF to only a small number of facilities. Further study is required to evaluate the utility of atrial pacing in PAF prevention.

## Treating PAF

PAF is typically transient and often requires no treatment. However, therapeutic intervention is needed in patients with compromised heart function, duration of AF >48 h, and high-risk patients with cerebral thromboembolism [37, 38, 61]. PAF can be treated by rhythm control, heart-rate control, and antithrombotic therapy, similar to the methods used for treating chronic AF. The Atrial Fibrillation Follow-Up Investigation of Rhythm Management (AFFIRM) study [62], which compared multiple methods of treatment for chronic AF, and the Rate Control versus Electrical (RACE) cardioversion for persistent atrial fibrillation trial [63] found that rate control was not inferior to rhythm control for preventing death and morbidity because antiarrhythmic agents used for rhythm control are associated with various adverse effects, including cardiac depression and a paradoxical increase in dangerous arrhythmias. Rate control with pharmacologic agents (either a β-blocker or a nondihydropyridine calcium-channel antagonist, in most cases) is recommended for patients with persistent or permanent AF. In the acute setting, intravenous administration of β-blockers (esmolol, metoprolol, or propranolol) or nondihydropyridine calcium-channel antagonists (verapamil, diltiazem) and amiodarone is recommended in order to slow the ventricular response to AF, exercising caution in patients with hypotension or heart failure [64]. β-blockers are more effective than calcium-channel antagonists for controlling the ventricular response during AF [65]. Further, β-blockers accelerate the conversion of postoperative supraventricular arrhythmias to sinus rhythm when compared with calcium-channel antagonists [21]. Cardioversion of AF is generally not recommended for asymptomatic or minimally symptomatic arrhythmias until the underlying problem is corrected. Indeed, correcting the underlying problem alone frequently leads to a spontaneous return to normal sinus rhythm (Class I, LOE A) [37]. In patients with stable hemodynamics, heart rate maintenance therapy is recommended. β-blockers, calcium-channel antagonists, and amiodarone are used to control heart rate. The target heart rate is 60–80/min at rest and 90–115/min during exercise. During the perioperative period, it is advisable to control heart rate at 90–115/min, similar to the range recommended for rate control during exercise [37].

PAF results in loss of the “atrial kick” and a 20–30% reduction in cardiac output. In patients with unstable hemodynamics (often seen after cardiovascular surgery), sinus rhythm is advantageous, and maintaining or restoring sinus rhythm is recommended [37]. It is recommend to restore sinus rhythm by pharmacologic cardioversion with ibutilide or via direct current cardioversion in patients who develop postoperative AF (Class IIa, LOE B) [37]. In the highly symptomatic patient or when rate control is difficult to achieve, cardioversion may be performed using the same precautions regarding anticoagulation as in nonsurgical cases. A variety of pharmacologic agents, including amiodarone, procainamide, ibutilide, and sotalol, may be effective to convert AF to sinus rhythm. Although a Class III agent was more effective than placebo for postoperative AF treatment in one study, orally administered sotalol is appealing in this situation because its β-blocking action slows the ventricular rate and its proarrhythmic toxicity is relatively low. However, this agent seems less effective than others for AF conversion. Otherwise, flecainide, dofetilide, ibutilide, propafenone (Class I, LOE A), and amiodarone (Class IIa, LOE A) are recommended for pharmacologic cardioversion. If these drugs are not effective, direct-current cardioversion can be performed. Antiarrhythmic medications should also be administered in an attempt to maintain sinus rhythm in patients with recurrent or refractory PAF. Dofetilide (Class I, LOE A) and amiodarone and ibutilide (Class IIa, LOE A) are recommended for maintaining sinus rhythm [37].

PAF is associated with an increased risk of cardiovascular and cerebrovascular events, especially strokes [3, 66]. Antithrombotic therapy is used in addition to heart-rate maintenance therapy in cases of PAF >48-h duration or in cases with a history of cerebrovascular thromboembolism. In contrast, anticoagulation in the perioperative period might increase the risk of bleeding or cardiac tamponade [67]. Antithrombotic agent selection should be based upon the absolute risks of stroke and bleeding and the relative risk and benefit for a given patient. Anticoagulation is more effective than aspirin to prevent stroke in patients with AF, as suggested by indirect comparisons and by a 33% risk reduction (95% CI 0.13–0.49) in a meta-analysis. Randomized trials involving high-risk AF patients (stroke rates >6% per year) show larger relative risk reductions in response to adjusted-dose orally administered anticoagulation therapy relative to aspirin, whereas the relative risk reductions are consistently smaller in trials of AF patients with lower stroke rates [68]. Thus, the first choice for antithrombotic therapy is anticoagulation. In those with contraindications to orally administered anticoagulation therapy, aspirin 81–325 mg daily is recommended as an alternative to vitamin K antagonists [37]. One meta-analysis reported that adjusted-dose orally administered anticoagulation threapy is highly efficacious for stroke prevention, with a risk reduction of 62% (95% CI 0.48–0.72) versus placebo in an intention to treat analysis [68]. Protection against stroke due to AF is achieved at a prothrombin-time international normalized ratio (PT-INR) range of 2.0–3.0 [69]. For patients without mechanical heart valves who are at high risk of stroke, chronic anticoagulant therapy orally with a vitamin K antagonist is recommended in a dose-adjusted manner to achieve the target intensity PT-INR of 2.0–3.0, unless contraindicated [69]. Factors associated with highest risk for stroke in patients with AF are prior thromboembolism (e.g., stroke, transient ischemic attacks, or systemic embolism) and rheumatic mitral stenosis (Class I, LOE A) [37]. Although the optimal duration of antithrombotic therapy has not been established, in general, if normal sinus rhythm returns, anticoagulation therapy can be stopped because its risks outweigh the benefits [61]. The risk of bleeding is higher in elderly patients receiving antithrombotic therapy. PT-INR should be controlled to 1.6–2.6 in patients ≥75 years of age [37]. In the clinical setting, anticoagulation therapy must be started when the risk–benefit ratio is favorable, especially in high-risk patients (e.g., elderly patients or those with uncontrolled hypertension or a history of bleeding). In the recent Randomized Evaluation of Long-Term Anticoagulation Therapy (RE-LY) trial, dabigatran administered at a dose of 150 mg was associated with lower rates of stroke and systemic embolism (relative risk 0.66; 95% CI 0.53–0.82; *p* < 0.001), but a similar rate of major hemorrhage (relative risk 0.93; 95% CI 0.81–1.07; *p* = 0.31) when compared with warfarin [70]. Although dabigatran is not a vitamin K antagonist, it is a potent, direct, competitive inhibitor of thrombin [70]. Another advantage of dabigatran is its lack of interactions with food and drugs. In addition, dabigatran does not require regular monitoring, and it has a quick onset of action and a short serum half-life [71]. In the future, use of dabigatran for perioperative management of PAF is expected to increase.

The impact of PAF on patient outcomes has prompted intense investigation into the optimal methods for prevention and treatment of this complication. PAF prevention is an important goal and requires specific perioperative management as well as other approaches. PAF is also associated with obesity, diabetes mellitus, and metabolic syndrome, which emphasizes the ongoing need for appropriate lifestyle management in individual patients.
